# Effectiveness of protocolized management for patients sustaining maxillofacial fracture with massive oronasal bleeding: a single-center experience

**DOI:** 10.1186/s13049-022-01047-9

**Published:** 2022-11-21

**Authors:** Fang-Chi Wu, Kuo-Shu Hung, Yu-Wen Lin, Kang Sung, Tsung-Han Yang, Chun-Hsien Wu, Chih-Jung Wang, Yi-Ting Yen

**Affiliations:** 1 Division of General Surgery, Department of Surgery, National Cheng Kung University Hospital, Tainan, Taiwan; 2 School of Medicine, College of Medicine, National Cheng Kung University, Tainan, Taiwan; 3 Department of Medical Imaging, National Cheng Kung University Hospital, Tainan, Taiwan; 4 Division of Trauma, Department of Surgery, National Cheng Kung University Hospital, College of Medicine, National Cheng Kung University, No. 138, Sheng Li Road, Tainan, 704 Taiwan

**Keywords:** Protocol, Oronasal bleeding, Maxillofacial fracture, Maxillofacial bleeding, Angiography, Arterial embolization

## Abstract

**Background:**

Maxillofacial fractures can lead to massive oronasal bleeding; however, surgical hemostasis and packing procedures can be challenging owing to complex facial anatomy. Only a few studies investigated maxillofacial fractures with massive oronasal hemorrhage. However, thus far, no studies have reported a protocolized management approach for maxillofacial trauma from a single center. This study aimed to evaluate the effectiveness of protocolized management for maxillofacial fractures with oronasal bleeding.

**Methods:**

Patients were identified from the National Cheng University Hospital trauma registry from 2010 to 2020. We included patients with a face Abbreviated Injury Scale (AIS) score of > 3 and active oronasal bleeding. Patients’ characteristics were compared between the angiography and non-angiography groups and between survivors and nonsurvivors.

**Results:**

Forty-nine patients were included. Among them, 34 (69%) underwent angiography, of whom 21 received arterial embolization. Forty-seven patients (96%) successfully achieved hemostasis by adhering to the treatment protocol at our institution. Compared with the non-angiography group, the angiography group had significantly more patients requiring oral intubation (97% vs. 53%, *P* < 0.001), Glasgow Coma Scale < 9 (GCS; 79% vs. 27%, *P* < 0.001), head AIS > 3 (65% vs. 13%, *P* = 0.001), higher Injury Severity Score (ISS; 43 [33–50] vs. 22 [18–27], *P* < 0.001), higher incidence of cardiopulmonary resuscitation (CPR; 41% vs. 0%, *P* = 0.002), higher mortality rate (35% vs. 7%, *P* = 0.043), and more units of packed red blood cells (PRBC) transfused within 24 h (12 [6–20] vs. 2 [0–4], *P* < 0.001). The nonsurvivor group had significantly more patients with hypotension (62% vs. 8%; *P* < 0.001), higher need for CPR (85% vs. 8%; *P* < 0.001), head AIS > 3 (92% vs. 33%; *P* < 0.001), skull base fracture (100% vs. 64%; *P* = 0.011), GCS score < 9 (100% vs. 50%; *P* = 0.003), higher ISS (50 [43–57] vs. 29 [19–48]; *P* < 0.001), and more units of PRBC transfused within 24 h (18 [13–22] vs. 6 [2–12]; *P* = 0.001) than the survivor group. More patients underwent angiography in the nonsurvivor group than in the survivor group (92% vs. 61%; *P* = 0.043). Among embolized vessels, the internal maxillary artery (65%) was the most common bleeding site. Hypoxic encephalopathy accounted for 92% of deaths.

**Conclusions:**

Protocol-guided management effectively optimizes outcomes in patients with maxillofacial bleeding.

## Background

Blunt craniofacial trauma with uncontrolled oronasal bleeding can result in hypovolemic shock, and the developed large blood clots may induce blockages in the airway. Moreover, the risk of airway obstruction is significantly higher if the patient has diminished consciousness after a head injury that frequently accompanies maxillofacial fractures [1, 2]. Respiratory arrest and even cardiac arrest may occur if a definitive airway is not established immediately.

Life-threatening hemorrhage secondary to maxillofacial trauma can rarely occur, but it can be fatal, with an approximate incidence of 1.2%–4.5% and a mortality rate of 20.21% [3–5]. Extensive collateral circulation in the face makes it difficult to identify the exact bleeders and subsequently delays hemorrhage control. Owing to the complexity of the facial anatomy, surgical hemostasis and packing procedures can be challenging to perform.

There are several measures to achieve successful hemostasis for oronasal bleeding, such as oronasal packing, balloon tamponade, electrocauterization, and suture ligation [6–11]. With advances in endovascular techniques, transarterial embolization (TAE) has emerged as an alternative to surgical ligation for maxillofacial bleeding control in many institutions since 2003 [3, 6, 10, 12, 13]. This less invasive endovascular procedure can effectively achieve hemostasis for oronasal bleeding; however, it is invasive and resource consuming. Moreover, clarifying the timing and indication of TAE is necessary to effectively use the limited resource when salvaging bleeding in patients. However, owing to the rare occurrence of maxillofacial bleeding, no standardized management protocol implementing TAE for hemostasis has been reported and practiced in a single institution.

Ten years ago, our institution established a protocol adopting TAE as the primary choice for intractable oronasal bleeding. Therefore, this present study aimed to evaluate the effectiveness of protocolized management of patients sustaining maxillofacial trauma with oronasal bleeding during a 10-year period.

## Methods

### Patient selection and evaluation

This retrospective study was approved by the institutional review board of National Cheng University Hospital (NCKUH) (B-ER-111-124). The requirement for informed consent was waived. The NCKUH trauma registry was queried from January 1, 2010 to December 31, 2020 to screen patients with a face Abbreviated Injury Scale (AIS) score of > 3. Specifically, patients with active oronasal bleeding were identified by reviewing the electronic medical records. Patients without significant oronasal bleeding, with incomplete data, and who were dead on arrival were excluded from the current study. Patients with significant concurrent active bleeding from the torso or extremities were also excluded from the study.

Demographic and clinical data included information on age; sex; initial emergency department systolic blood pressure (SBP); cardiopulmonary resuscitation (CPR); mechanism of injury; emergency airway management; transfusion requirements within the first 24 h; Injury Severity Score (ISS); initial Glasgow Coma Scale (GCS) score; hospital length of stay (LOS); and rates of intensive care unit (ICU) admission, in-hospital mortality, and concomitant injuries.

### Protocol for maxillofacial trauma

**Fig. 1:**
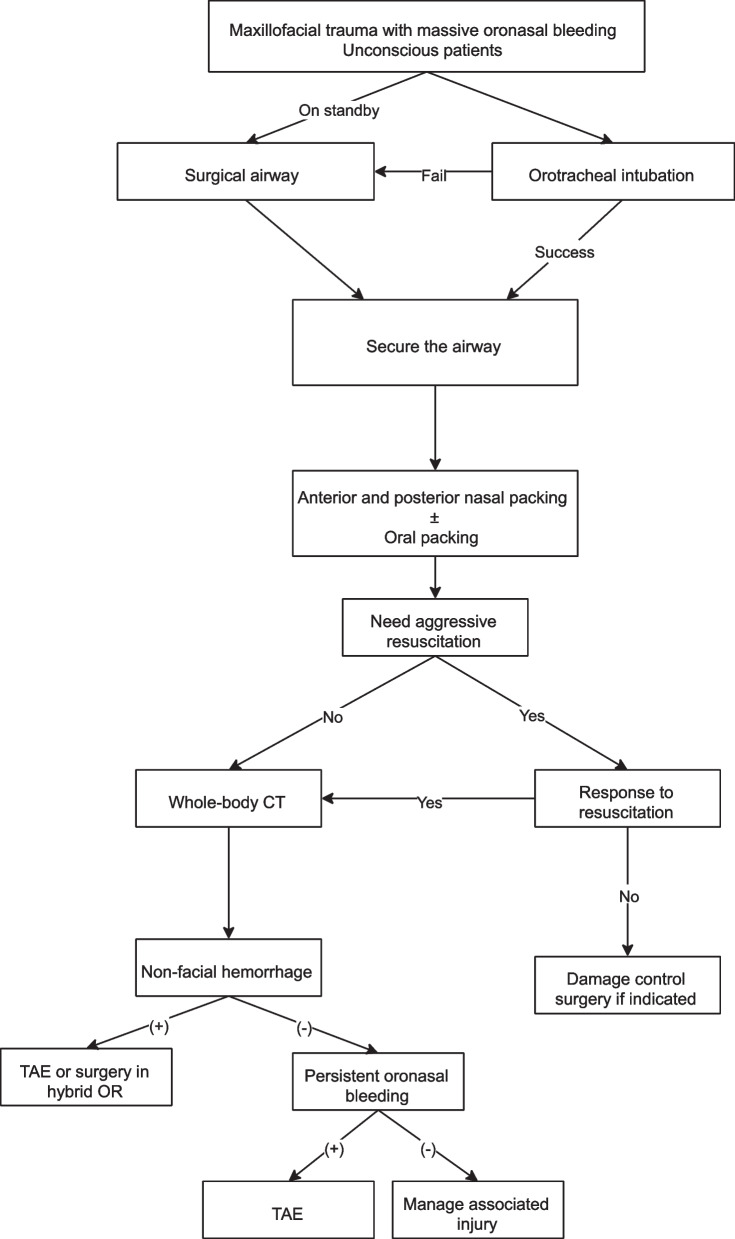
Management algorithm

NCKUH is a tertiary referral medical center with a 1200-bed capacity. The trauma team was established in 2010 to provide critical care to patients who sustained traumatic injuries. A protocol was established to manage patients sustaining maxillofacial trauma with oronasal bleeding (Fig. 1).

For patients with active oronasal bleeding, evaluation and establishment of a patent airway were the first steps. When orotracheal intubation fails, surgical airways, such as cricothyrotomy or tracheostomy, are attempted. Following a secure airway, anterior nasal packing with gauze and posterior nasal packing with Foley catheter balloon tamponade were suggested as initial attempts to achieve hemostasis. Oral packing was performed if blood gushed out of the mouth. Emergency angiography was indicated for patients with persistent oronasal bleeding after packing or for those who still needed aggressive blood transfusion without other identified active bleeding sources. For patients who required TAE for concomitant abdominal or other bleeding, inspection for possible bleeding in the maxillofacial region during the same session was suggested. After angiographic embolization, patients were monitored in the ICU. Secondary angiography was indicated in consideration of delayed or persistent bleeding.

### Statistical analysis

Continuous variables are described as median and interquartile ranges, and categorical data are described as counts and percentages. Differences in categorical values between groups were compared using χ^2^ analysis or Fisher’s exact test, while Mann–Whitney U or Kruskal–Wallis tests were used to examine differences in continuous variables. Differences were considered statistically significant at *P* < 0.05. All data analyses were performed using IBM SPSS software (version 18.0, Chicago, IL, USA).

## Results

### Patient population

**Fig. 2:**
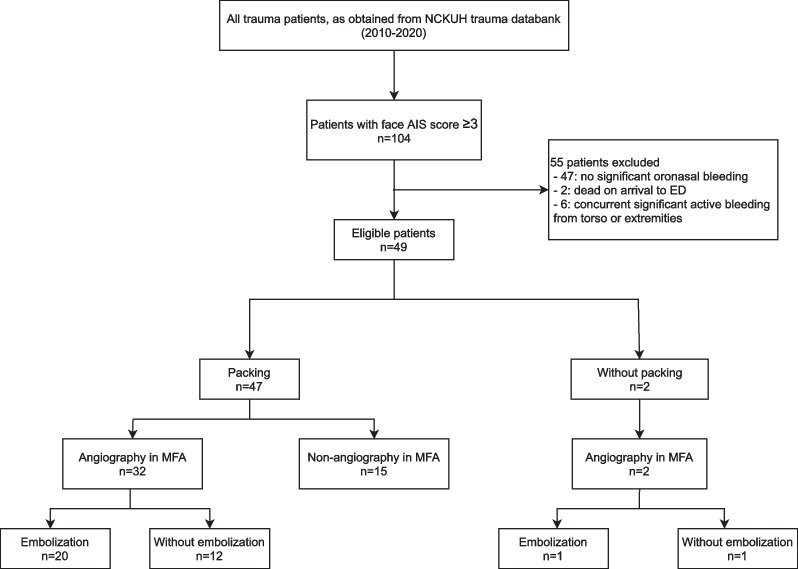
Patient selection process and management

Figure 2 shows the patient selection process. A total of 104 patients with a facial AIS of > 3 were identified during the 10-year period. After excluding 55 patients, 49 were included in the current study.

### Demographics

Fifteen patients (31%) achieved hemostasis by oronasal packing alone without the need for further angiography. Thirty-four patients (69%) underwent angiography, of whom 21 received arterial embolization. Forty-seven patients (96%) successfully achieved hemostasis after oronasal packing alone or combined with TAE. Two patients failed to achieve hemostasis. One patient had an inaccessible post-traumatic skull base arteriovenous (AV) fistula with active bleeding, and the other patient had sudden cardiac arrest during TAE. Only one patient required a second embolization to achieve complete hemostasis. Regarding bleeding propensity, only one patient received antiplatelets before trauma. This patient underwent embolization and survived.

### Comparison of the angiography and non-angiography groups

The majority of patients were male (80%), and the median age was 31 (19–57) years old. The major mechanism of injury was traffic accident (80%). Compared with the non-angiography group (NAG), the angiography group (AG) had significantly more patients requiring oral intubation (97% vs. 53%, *P* < 0.001), more patients with GCS < 9 (79% vs. 27%, *P* < 0.001), head AIS > 3 (65% vs. 13%, *P* = 0.001), higher ISS (43 [33–50] vs. 22 [18–27], *P* < 0.001), higher incidence of CPR (41% vs. 0%, *P* = 0.002), higher mortality rate (35% vs. 7%, *P* = 0.043), and more blood transfusions within 24 h (12 [6–20] vs. 2 [0–4], *P* < 0.001; Table 1).

**Table 1:** Demographics of patients with maxillofacial hemorrhage

	Total, N = 49	Angiography group, n = 34	Non-Angiography group, n = 15	*P*
Male	39 (80)	27 (79)	12 (80)	> 0.999
Age: median (IQR), y	32 (19–57)	30(19–60)	41.5 (21–49)	0.688
Mechanism of injury				0.186
Fall	6 (12)	6 (18)	0 (0)	0.159
Traffic accident	39 (80)	26(76)	13 (87)	0.702
Others	4 (8)	2 (6)	2 (13)	0.576
SBP				0.137
≥ 90	38 (76)	24 (70)	11 (92)	
< 90	11 (22)	10 (30)	1 (8)	
GCS score				< 0.001
3–8	31 (63)	27 (79)	4 (27)	
9–12	3 (6)	2 (6)	1 (7)	
13–15	15 (31)	5 (15)	10 (67)	
Emergency airway management	41 (84)	33 (97)	8 (53)	< 0.001
Emergency surgical airway	6 (12)	5 (15)	1 (7)	0.652
ISS: median (IQR)	38 (22–50)	43 (33–50)	22 (18–27)	< 0.001
Head AIS > 3	24 (49)	22 (65)	2 (13)	0.001
Chest AIS > 3	12 (25)	10 (29)	2 (13)	0.298
Abdomen AIS > 3	0 (0)	0 (0)	0 (0)	Null
Extremity AIS > 3	1 (2)	1 (3)	0 (0)	1.000
CPR	14 (29)	14 (41.2)	0 (0)	0.002
Intracranial vascular injury	5 (10)	5 (15)	0 (0)	0.306
Skull base fracture	36(74)	27 (80)	9 (60)	0.178
PRBC within 24 h after arrival at ER: median (IQR), units	10 (3–18)	12 (6–20)	2 (0–4)	< 0.001
ICU admission rate	37 (76)	31 (91)	6 (40)	< 0.001
LOS: median (IQR), day	14 (6–25)	15 (6–31)	14 (5–17)	0.409
Pre-injury antiplatelets use	1 (2)	1 (3)	0 (0)	0.694
Mortality	13 (27)	12 (35)	1 (7)	0.043

*SBP* systolic blood pressure, *GCS* Glasgow Coma Scale, *PRBC* packed red blood cells, *IQR* interquartile range; Traffic accidents indicate car, motorcycle, or bicycle accidents, *LOS* length of stay

There were no significant differences between the two groups with regard to age, sex, or mechanism of injury. The proportion of patients with SBP < 90 mmHg, skull base fracture, intracranial vascular injury, and length of hospital stay was similar between the two groups.

### Comparison of survivors and nonsurvivors

Table 2 shows the characteristics of the survivors and non-survivors. Thirty-six patients (73%) survived until discharge, whereas 13 patients (27%) died. There were no significant differences between the two groups with regard to age, sex, or mechanism of injury.

**Table 2:** Comparison of survivors and nonsurvivors

	Survivors, n = 36	Nonsurvivors, n = 13	*P*
Male	29 (81)	10 (77)	> 0.999
Age: median (IQR), y	33 (19–59)	29 (18–50)	0.454
Mechanism of injury			0.606
Fall	5 (14)	1 (8)	> 0.999
Traffic accident	27 (75)	12 (92)	0.253
Others	4 (11)	0 (0)	0.562
SBP			< 0.001
≥ 90	34 (94)	5 (38)	
< 90	2 (6)	8 (62)	
GCS score			0.003
3–8	18 (50)	13 (100)	
9–12	3 (8)	0 (0)	
13–15	15 (42)	0 (0)	
Emergency airway management	28 (78)	13 (100)	0.09
Emergency surgical airway	3 (8)	3 (23)	0.321
ISS: median(IQR)	29 (19–48)	50(43–57)	< 0.001
Head AIS > 3	12 (33)	12 (92)	< 0.001
Chest AIS > 3	7 (19)	5 (39)	0.259
Abdomen AIS > 3	0 (0)	0 (0)	NULL
Extremity AIS > 3	1 (3)	0 (0)	> 0.999
CPR	3 (8)	11 (85)	< 0.001
Intracranial vascular injury	2 (6)	3 (23)	0.109
Skull base fracture	23 (64)	13 (100)	0.011
Intervention: angiography	22 (61)	12 (92)	0.043
PRBC within 24 h after arrival at ED: median (IQR), units	6 (2–12)	18 (13–22)	0.001
ICU admission rate	25 (69)	12 (92)	0.142
Length of stay: median (IQR)	20 (12–31)	5 (1–9)	< 0.001
Pre-injury antiplatelets use	1 (3)	0 (0)	0.735

*IQR* interquartile range; Traffic accidents include car, motorcycle, or bicycle accidents

The non-survivor group had significantly more patients with hypotension (62% vs. 8%; *P* < 0.001), higher incidence of CPR (85% vs. 8%; *P* < 0.001), and more units of packed red blood cells (PRBC) transfused within 24 h (18 [13–22] vs. 6 [2–12]; *P* = 0.001) than the survivor group. Non-survivors were associated with higher incidence of head AIS > 3 (92% vs. 33%; *P* < 0.001), skull base fracture (100% vs. 64%; *P*  = 0.011), GCS < 9 (100% vs. 50%; *P*  = 0.003), and higher ISS (50 [43–57] vs. 29 [19–48]; *P* < 0.001) compared with survivors. Additionally, more patients in the non-survivor group underwent angiography (92% vs. 61%; *P*  = 0.043).

### Description of mortality

Table 3 describes the characteristics of non-survivors. The hospital LOS ranged from 1 to 16 days. Four patients died on day 1, in whom the main causes of death were as follows: (1) inaccessible posttraumatic AV fistula with persistent bleeding (one patient), (2) sudden cardiac arrest (one patient), and (3) hypoxic encephalopathy (two patients). Among non-survivors, two patients required more than 6 units of PRBC transfusion within 24 h after TAE. Twelve patients (92%) had hypoxic encephalopathy.

**Table 3:** Characteristics of non-survivors

Patient No	Initial GCS	LOS	Head AIS	Oronasal packing	Angiography	Complete Embolization	Inaccessible lesions	Blood transfusion > 6Uwithin 24 h after angiography	Main cause of death
1	3	1	5	Yes	Yes	No	Small caliber hemorrhaging a	8U	Hypoxic encephalopathy
2	3	5	5	Yes	Yes	Yes		No	Hypoxic encephalopathy
3	3	16	5	Yes	Yes	Yes		No	Hypoxic encephalopathy
4	2 T	10	5	Yes	Yes	No	No obvious contrast extravasation (ICA pseudoaneurysm)	No	Hypoxic encephalopathy
5	2 T	9	5	Yes	Yes	No	Small caliber hemorrhaging a	No	Hypoxic encephalopathy
6	3	1	5	Yes	Yes	No	Skull base AV fistula with active bleeding with active bleeding	18U	Hypoxic encephalopathy and bleeding
7	7	12	5	YES	YES	NO	Left ACA ruptured pseudoaneurysm	No	Hypoxic encephalopathy
8	3	4	5	Yes	Yes	No	No obvious contrast extravasation	No	Hypoxic encephalopathy
9	3	1	5	No	Yes	Yes		No	Hypoxic encephalopathy
10	2 T	5	5	Yes	No	No		No	Hypoxic encephalopathy
11	3	6	5	No	Yes	No	No obvious contrast extravasation	No	Hypoxic encephalopathy
12	3	1	3	Yes	Yes	No	CPR during TAE	No	Bleeding from left neck area
13	3	2	5	Yes	Yes	No	Small caliber hemorrhaging a	No	Hypoxic encephalopathy

### Embolized vessels

**Fig. 3:**
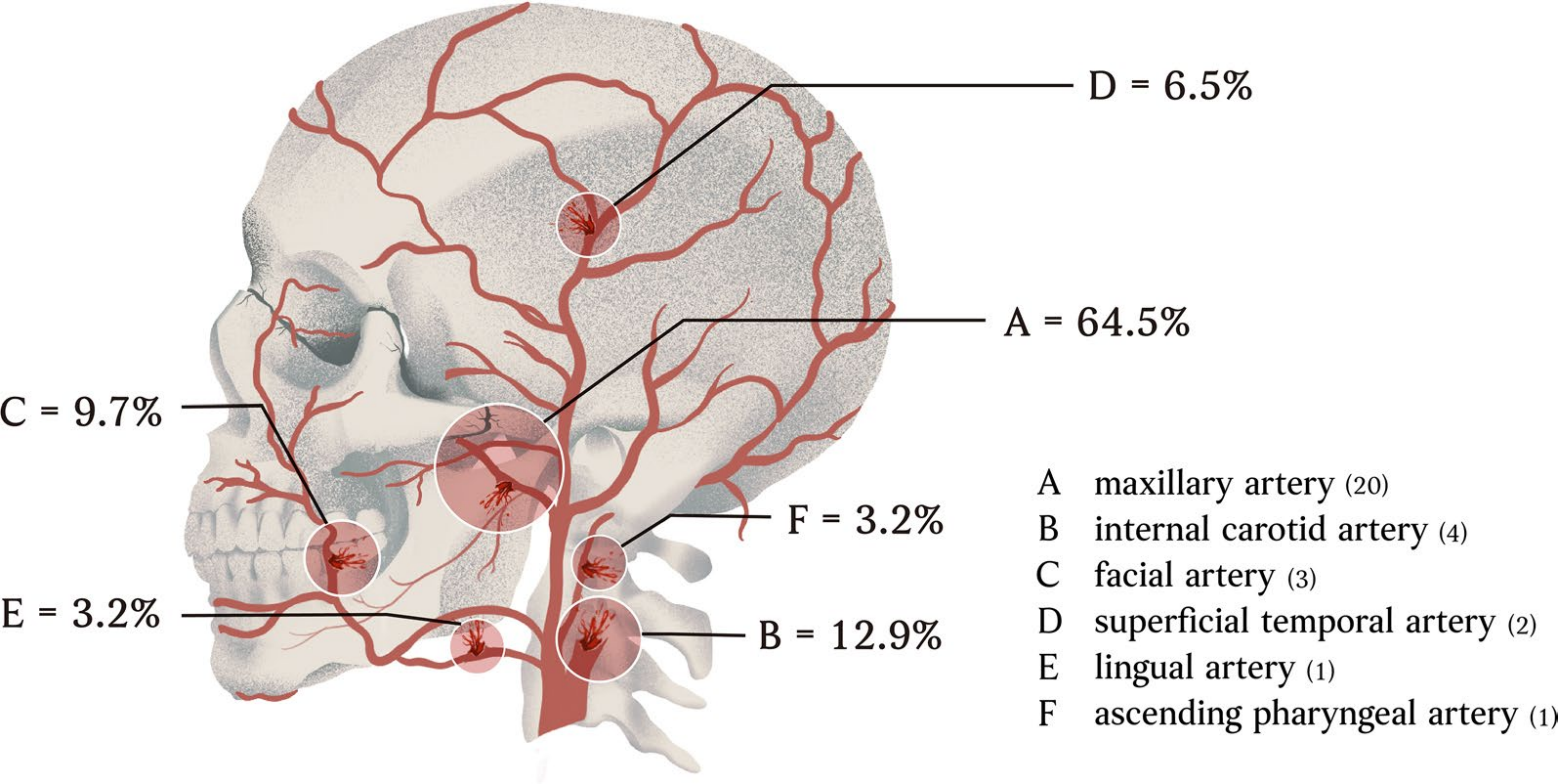
Thirty-one vessels treated by TAE

Thirty-one arteries were embolized in 21 patients. The most common embolized arteries were the internal maxillary artery (n = 20, 64.5%), followed by the branches of the internal carotid artery (n = 4, 12.9%) and the facial artery (n = 3, 9.7%) (Fig. 3).

## Discussion

Our study showed that oronasal packing combined with TAE successfully achieved hemostasis in 47 patients (96%), and 36 patients (73%) survived by adhering to the treatment protocol at our institution. Only two patients (4%) died of persistent bleeding from inaccessible lesions by angiography in the present study. Protocol-guided management of maxillofacial trauma-related oronasal bleeding was effective in achieving hemostasis in most patients. To the best of our knowledge, this is the largest single-center report on the management of maxillofacial trauma with oronasal bleeding (Table 4).

**Table 4:** Thirty-one vessels treated by TAE

Internal maxillary artery	20
Superficial temporal artery	2
Facial artery	3
Ascending pharyngeal artery	1
Lingual artery	1
The internal carotid artery and its branches^a^	4

^a^Anterior cerebral artery pseudoaneurysm, ICA pseudoaneurysm, ICA injury with contrast extravasation and ethmoid artery

Hypoxic encephalopathy was the main cause of death in patients with massive oronasal bleeding. The current study showed that 92% of deaths were caused by hypoxic encephalopathy, and 85% of deaths occurred because of cardiac arrest related to a compromised airway. Securing the airway is the top priority for saving maxillofacial trauma patients with active oronasal bleeding, especially those who are unconscious [6, 7, 13, 14]. Additionally, timely management may reduce the consequences of hypoxic injury, such as death, prolonged coma, permanent cognitive deficits, seizures, and other neurological abnormalities. In the face of active oronasal bleeding, trauma surgeons should not hesitate to establish a surgical airway when the first attempt of orotracheal intubation fails. Moreover, prehospital intubation with an intent to prevent blood from obscuring the airway may decrease the incidence of hypoxia and aspiration, which may be associated with better neurological outcomes and reduced in-hospital mortality [15, 16].

Anterior and posterior nasal packing, balloon tamponade, and conservative measures have been proposed as the first attempts to manage oronasal hemorrhage [6–11]. Anterior and posterior nasal packing were used to apply pressure on the Kiesselbach's plexus and the Woodruff plexus, respectively. Cogbill et al. reported that anterior, posterior, or both packing controlled the bleeding in only 29% of patients and slowed the rate in 44% of patients [6]. This finding is comparable with that of the current study, in which hemostasis was achieved in 15 patients (30%) by oronasal packing alone. However, disruption of facial buttresses and extensive collateral circulation between the external and internal carotid artery systems may render packing and tamponade less effective in hemostasis [17]. External carotid artery (ECA) ligation has been recommended in the literature if oronasal bleeding continues despite packing. ECA ligation is rarely effective in controlling such hemorrhage because of the abundant collateral circulation of the face [18]. TAE can precisely identify and stop bleeding with few complications. TAE has emerged as the primary choice for hemostasis in patients with persistent bleeding after packing [3, 6, 10, 12, 13]. In this study, 34 patients (69%) proceeded to angiography for persistent bleeding after packing; among them, 22 patients (65%) were salvaged by TAE.

The advantages of TAE over ligation include shorter procedure time, more precise hemorrhage localization, and the ability to embolize a possible concomitant abdominal or other bleeding during the same session [10, 19]. Based on a review of 205 cases, the efficacy of TAE was 79.4%–100%, while the rate of major complications was approximately 2%–4% [20]. In our study, 34 patients underwent TAE, and definitive control of hemorrhage was achieved in 32 patients (94%), which is consistent with the finding reported in the literature. The internal maxillary artery and its branches are most frequently associated with traumatic maxillofacial hemorrhage [3, 6, 8, 10, 12].

The proper choice of embolic agents is crucial for interventional radiologists to avoid serious neurological complications, such as cerebrovascular accidents, cranial nerve palsy, and brain infarction. Microcoils and gelfoam pledgets are the embolic agents most frequently used for TAE in maxillofacial trauma [20]. Embolic agents such as gelfoam may pass through the extracranial–intracranial anastomoses, consequently causing embolic complications [21]. No major procedure-related complications were observed in our study.

Embolization can be used to prevent bleeding. However, the approach to craniofacial bleeding has some limitations. Bleeding from the ICA and its branches should be treated carefully with TAE in the case of passage of embolic agents into the brain, leading to neurological injury. Four bleeding sources in the ICA or its branches were noted in our study: ruptured ACA pseudoaneurysm, unruptured ICA pseudoaneurysm, bilateral ICA laceration, and ethmoidal artery.

Coils were used in the ruptured ACA pseudoaneurysm, but they only partially reduced the bleeding because of the difficulty in accessing the lesion. No embolization was performed in the unruptured ICA pseudoaneurysm because of the absence of contrast extravasation. Active bleeding from the bilateral ICA laceration was identified; therefore, coils were used, and bleeding was successfully arrested. A low flow rate of contrast extravasation was identified in the ethmoidal artery; therefore, embolization was not performed.

It is worth noting that arterial or venous bleeding from oral and nasal mucosa might continue after packing and arterial embolization. Therefore, our protocol suggests that packing should be performed for 48–72 h until hemostasis is confirmed. A second angiography can be performed if delayed or persistent bleeding occurs. Seven patients received a PRBC transfusion of more than 6 units 24 h after angiography. Only one of those patients underwent a second angiography after receiving 20 units of PRBC transfusion. No contrast extravasation in the maxillofacial area was identified in the second angiography. Owing to persistent bleeding from the oronasal mucosa found on examination, prolonged packing and suture ligation were performed, and the patient survived after successful hemostasis. Four patients achieved hemostasis with prolonged packing and survived. One patient died of persistent bleeding from the skull base AV fistula, which was not embolized owing to its inaccessibility. One patient achieved hemostasis by packing but died of hypoxic encephalopathy.

Matsumoto et al. selected cases of Le Fort III fractures with blood loss greater than 20% from Japan trauma data bank and reported that the in-hospital mortality of the TAE group was significantly lower than that of the non-TAE group (23.1% vs. 44.6%; *P* = 0.048) [13]. Our study found a similar result that TAE can effectively arrest bleeding. However, the angiography group showed a significantly higher mortality rate (35%). Our study showed that the angiography group had significantly lower GCS and higher ISS scores. Therefore, the higher mortality rate was more likely to result from the patient-associated injury than TAE.

Intracranial injury or hemorrhage is the most common type of concomitant injury in patients with panfacial fractures [1, 2]. In this study, 49% of the included patients had a head AIS score of > 3. Bromberg et al. [22] reported that blunt trauma patients with arterial hemorrhage from neck/nose/mouth and midface fractures (Le Fort II or III) are at an increased risk of blunt cerebrovascular injury (BCVI), which might lead to permanent severe neurologic deficits in survivors. This study identified five cerebrovascular injuries after trauma, including carotid-cavernous fistula (two patients), ACA pseudoaneurysm (one patient), ICA pseudoaneurysm (one patient), and skull base AV fistula (one patient) in the angiography group. The incidence of BCVI was 15% in the angiography group. Two patients with carotid-cavernous fistulas survived with conservative management and did not develop severe neurologic deficits. Two patients with a ruptured ACA pseudoaneurysm and skull base AV fistula died due to persistent bleeding. The endovascular intervention failed to access the lesion and arrest the bleeding. Although no active bleeding was identified in the ICA pseudoaneurysm, the patient died of hypoxic encephalopathy.

### Limitations

This study has several limitations. This study was a retrospective analysis of the data. First, the number of patients identified in this single-center study was small. The timing of TAE depended on clinical judgment, which may have influenced the outcome. Moreover, the selective or nonselective embolization techniques and the choice of embolic agents depended on the clinical assessment of the interventional radiologists. This variation could also affect the outcomes. During the study period, some changes were made in the treatment of bleeding trauma patients. In 2013, 1 g tranexamic acid was adopted in the emergency department and the massive transfusion protocol was established in 2015 as standard care for bleeding trauma patients. Both may affect clinical outcomes. However, because of the small number of patients, no survival benefit was observed with these two changes. Multi-institutional prospective studies are needed to further this research.

## Conclusions

Severe oronasal hemorrhage secondary to maxillofacial fractures is lethal. Securing the airway and early hemostasis is the top priority for saving such patients. Balloon tamponade and oronasal packing, followed by TAE, effectively achieved hemostasis. Protocol-guided management was effective in optimizing outcomes in patients with maxillofacial bleeding. Future multi-institutional prospective studies are needed to assess the effectiveness of protocol-guided management.

## Data Availability

The datasets used and/or analyzed during the current study are available from the corresponding author on reasonable request.

## Abbreviations

NCKUH: National Cheng Kung University Hospital
TAE: Transcatheter arterial embolization
AIS: Abbreviated Injury Scale
CPR: Cardiopulmonary resuscitation
SBP: Systolic blood pressure
ISS: Injury Severity Score
GCS: Glasgow Coma Scale
LOS: Length of stay
ICU: Intensive care unit
NAG: Non-angiography group
AG: Angiography group
ECA: External carotid artery
BCVI: Blunt cerebrovascular injury
